# Discovery of novel SOS1 inhibitors using machine learning†

**DOI:** 10.1039/d4md00063c

**Published:** 2024-03-15

**Authors:** Lihui Duo, Yi Chen, Qiupei Liu, Zhangyi Ma, Amin Farjudian, Wan Yong Ho, Sze Shin Low, Jianfeng Ren, Jonathan D. Hirst, Hua Xie, Bencan Tang

**Affiliations:** a Nottingham Ningbo China Beacons of Excellence Research and Innovation Institute, Key Laboratory for Carbonaceous Waste Processing and Process Intensification Research of Zhejiang Province, Department of Chemical and Environmental Engineering, The University of Nottingham Ningbo China 199 Taikang East Road Ningbo 315100 P. R. China Jianfeng.Ren@nottingham.edu.cn Bencan.Tang@nottingham.edu.cn; b School of Chemistry, University of Nottingham University Park Nottingham NG7 2RD UK jonathan.hirst@nottingham.ac.uk; c Division of Antitumor Pharmacology, State Key Laboratory of Drug Research, Shanghai Institute of Materia Medica, Chinese Academy of Sciences 555 Zuchongzhi Road 201203 Shanghai China hxie@simm.ac.cn; d Faculty of Medicine and Health Sciences, University of Nottingham (Malaysia Campus) Semenyih 43500 Malaysia; e University of Chinese Academy of Sciences No.19A Yuquan Road Beijing 100049 China; f Zhongshan Institute for Drug Discovery, Shanghai Institute of Materia Medica, Chinese Academy of Sciences Zhongshan Tsuihang New District Zhongshan 528400 China; g School of Mathematics, Watson Building, University of Birmingham Edgbaston Birmingham B15 2TT UK

## Abstract

Overactivation of the rat sarcoma virus (RAS) signaling is responsible for 30% of all human malignancies. Son of sevenless 1 (SOS1), a crucial node in the RAS signaling pathway, could modulate RAS activation, offering a promising therapeutic strategy for RAS-driven cancers. Applying machine learning (ML)-based virtual screening (VS) on small-molecule databases, we selected a random forest (RF) regressor for its robustness and performance. Screening was performed with the L-series and EGFR-related datasets, and was extended to the Chinese National Compound Library (CNCL) with more than 1.4 million compounds. In addition to a series of documented SOS1-related molecules, we uncovered nine compounds that have an unexplored chemical framework and displayed inhibitory activity, with the most potent achieving more than 50% inhibition rate in the KRAS G12C/SOS1 PPI assay and an IC_50_ value in the proximity of 20 μg mL^−1^. Compared with the manner that known inhibitory agents bind to the target, hit compounds represented by **CL01545365** occupy a unique pocket in molecular docking. An *in silico* drug-likeness assessment suggested that the compound has moderately favorable drug-like properties and pharmacokinetic characteristics. Altogether, our findings strongly support that, characterized by the distinctive binding modes, the recognition of novel skeletons from the carboxylic acid series could be candidates for developing promising SOS1 inhibitors.

## Abstract 2

Machine learning enabled ligand-based virtual screening is a valuable tool in discovering effective SOS1 inhibitors.

## Graphical abstract

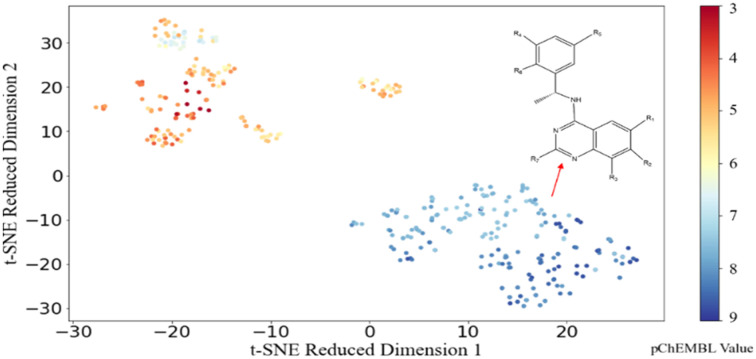

## 1. Introduction

**Fig. 1:**
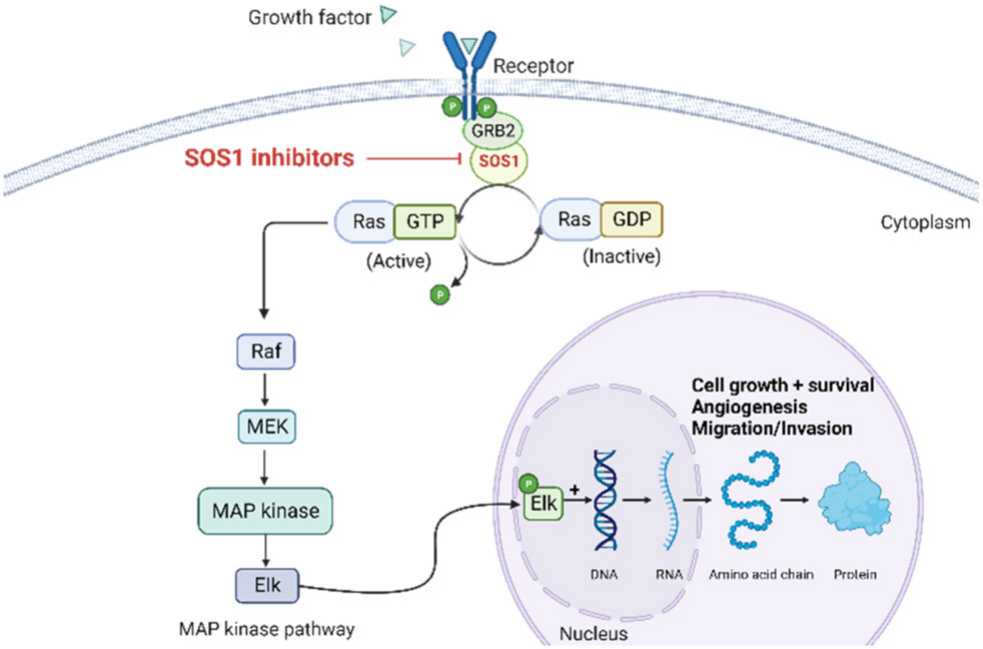
Mechanism of SOS1 inhibitors in RAS-driven malignancies. KRAS can exist in inactive (GDP-bound) or active (GTP-bound) states. SOS1 facilitates the exchange of GDP for GTP, activating RAS. In its activated state, RAS interacts with downstream effectors, initiating vital cellular pathways, such as MAP kinase signalling, critical for cell survival, growth, angiogenesis, cancer cell invasion, and migration. SOS1 inhibitors block GTP loading, maintaining RAS in an inactive state, preventing excessive downstream signalling, and offering a potential therapeutic approach for RAS-driven malignancies. Figure created with https://BioRender.com.

The rat sarcoma virus (RAS) superfamily members, including *HRAS*, *KRAS*, and *NRAS* in mammals, play significant roles in the pathogenesis of various human cancers.^1^ Notably, KRAS is commonly mutated, contributing to the activation of this gene in a multitude of cancer cases, including 80% to 90% of pancreatic cancers, 40% to 50% of colorectal cancers, and 30% of non-small cell lung cancers.^1^ However, the clinical therapeutic options are considerably constrained for individuals harbouring KRAS mutations. There are only two small-molecule inhibitors, sotorasib and adagrasib, currently approved by the FDA for treating the KRAS G12C mutated non-small cell lung cancer, indicating a significant unmet clinical need for KRAS-targeted therapies.^2,3^ As shown in Fig. 1, the mutation of KRAS is associated with the activation of multiple downstream signalling pathways, notably the RAF–MEK–ERK pathway, within the MAPK family, which are crucial for regulating cell survival and proliferation.^1,4^ The RAS proteins function as molecular switches, transitioning between the active on-state when bound to guanosine triphosphate (GTP) and the inactive off-state when bound to guanosine diphosphate (GDP).^5^ This switch is regulated by guanine nucleotide exchange factors, which promote the exchange of GDP for GTP, and GTPase-activating proteins, which enhance the hydrolysis of GTP to GDP.^2^ Son of sevenless 1 (SOS1), as a major guanine nucleotide exchange factor, plays a crucial role in the RAS signalling pathways by facilitating guanine nucleotide exchange and regulating KRAS switching from “GDP-bound off state” to “GTP-bound on state”.^6,7^ Hence, inhibiting the interaction between KRAS-GDP and SOS1 effectively reduces the formation of activated KRAS-GTP, thereby suppressing uncontrolled downstream cell proliferation.

**Fig. 2:**
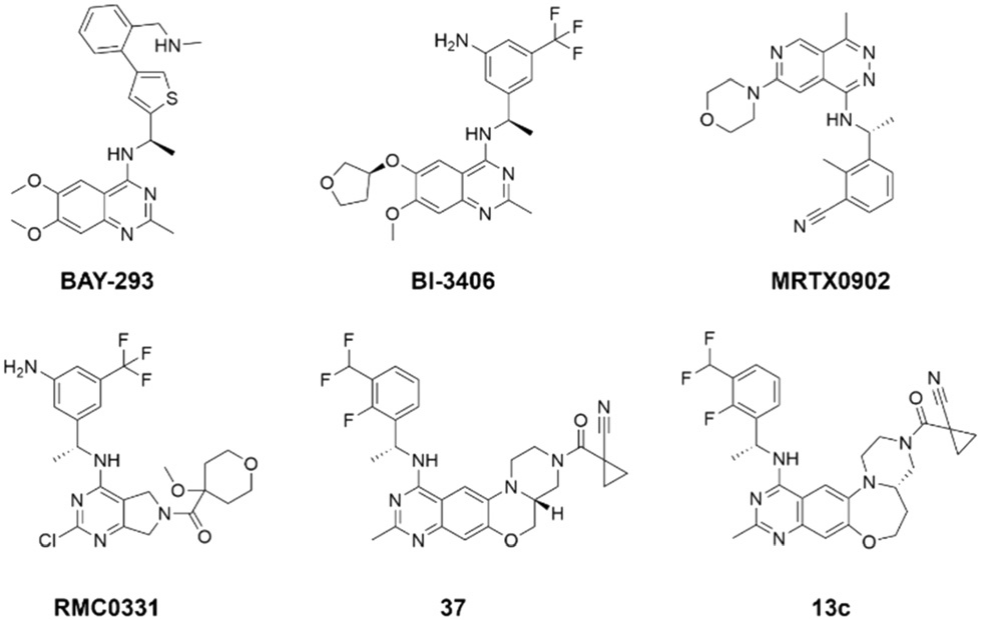
Representative SOS1 inhibitors.

Given the pivotal role of SOS1 in the progression of RAS-driven cancer, inhibiting the binding between SOS1 and RAS has emerged as a promising therapeutic avenue against RAS-driven tumours. Small molecule SOS1 inhibitors modulate RAS activation by binding to the SOS1 protein pocket, affecting the interaction between SOS1 and RAS.^8,9^Fig. 2 shows some examples of reported SOS1 compounds. For instance, Hillig *et al.* reported an aminoquinazoline inhibitor, **BAY-293**, based on fragment and high-throughput screening for the KRAS-SOS1 binding site.^10^ In 2021, Hofmann *et al.* discovered another aminoquinazoline compound, **BI-3406**, as an orally administered, selective, and highly potent SOS1 inhibitory agent.^11^ Its analogue, **BI 1701963** (whose structure has not been disclosed) was introduced into the first clinical study of SOS1 inhibitors but with a disappointing outcome. Another compound, **MRTX0902**, with a pyridopyridazine core, has just entered clinical trials (NCT05578092), which are designed to elucidate the effectiveness of **MRTX0902**, either alone or in combination with **MRTX849** (adagrasib), in treating solid tumours malignancies among patients harbouring KRAS G12C mutations.^12^ Similar research has been published by Revolution Medical^13^ and He *et al.*^14,15^ for the SOS1 inhibitors: **RMC-0331** with a pyrrolo [3,4-*d*]pyrimidine scaffold and the tetracyclicquinazoline (**37** and **13c**) with superior pharmacokinetic properties, respectively (see Fig. 2). Despite these promising advances, there is currently no approved SOS1 inhibitor, and most of these candidates are designed to be combined with anti-cancer drugs targeting the KRAS-MAP kinase pathway. Notably, most current SOS1 inhibitors are derived from **BAY-293** and lack structural diversity. Thus, there is an urgent need to identify and explore novel SOS1 inhibitors.

With the increased access to large-scale datasets, ML is revolutionizing drug development endeavours, especially for anti-cancer drug discovery, by offering the unparalleled potential to accelerate this process with higher success rates and lower cost compared with traditional pharmaceuticals.^16–18^ One common artificial intelligence (AI)-assisted drug discovery technique is virtual screening (VS), which is used to identify prospective leads from large compound libraries.^19^ Ligand-based virtual screening (LBVS) can be applied to target proteins with unknown structures to discover novel ligands based on the premise that substances with comparable structural properties are likely to exhibit comparable biological activities.^20^ Quantitative structure–activity relationships (QSAR), an important LBVS strategy, explore the correlation between chemical structures and biological activities of molecules to develop predictive models. Indeed, several such endeavours have been reported recently. Valentini *et al.* in 2022 utilized a comprehensive QSAR approach, integrating various ML techniques (K-nearest neighbour, gradient bosting, logistic regression, RF, and support vector machine), alongside *in vitro* and *in vivo* experimental data, to successfully identify two novel inhibitors of anti-apoptotic proteins with promising efficacy across multiple tumour histocytes.^21^ In another study, Yang *et al.* employed a combined approach of ligand-based ML and structural-based molecular docking to screen the latest US Food and Drug Administration approved drug library (∼2600 compounds) for potential inhibitors of adipocyte fatty acid-binding protein, seeking to investigate current medications with established safety characteristics.^22^ The results demonstrated the efficacy of the naïve Bayesian model in predicting potential inhibitors, leading to the discovery of cobimetinib, which was subsequently confirmed to inhibit A-FABP-activated JNK/C-jun phosphorylation in cellular assays. Other related research efforts are focused on the discovery of lead anti-cancer compounds such as lysine-specific histone demethylase 1,^23^ and indoleamine 2,3-dioxygenase inhibitors.^24^ All these initiatives highlight the tremendous promise that ML strategies hold in the field of drug discovery, particularly in the context of LBVS.

In summary, SOS1 is a crucial protein within the RAS pathway. Recently, targeting SOS1 has garnered increasing attention as a promising strategy for treating RAS-driven cancers. However, the development of effective and selective SOS1 inhibitors remains a challenge and an urgent demand. Thus, the aim of this research work is to use ML to predict the bioactivity of small molecules against SOS1 with a view to discovering structurally novel inhibitors.

## 2. Experimental

Our approach includes seven steps: 1) raw data collection from the ChEMBL dataset, including data curation, data cleaning and the calculation of molecular representations; 2) model development and optimization; 3) evaluation and validation of ten different ML models, selection of the best performing one; 4) VS of molecules from in-house libraries using the robust LBVS model to identify and rank hits; 5) biological experiments focusing on the KRAS G12C/SOS1 PPI assay; 6) molecular docking to analyse hits and receptor interactions; 7) *in silico* evaluation of drug-likeness properties.

### 2.1 Data collection and pre-processing

To compile the SOS1 training set, the publicly available ChEMBL database (version 31) was queried, focusing on records related to anthropogenic ligands tested against the SOS1 target (ChEMBL ID: CHEMBL2079846). The data were stored in the form of Simplified Molecular Input Line Entry System (SMILES) representations, along with various types of bioactivity measurements such as IC_50_, EC_50_, AC_50_, *F*_c_, *K*_i_, *E*_max_, and *K*_d_, reported in molar concentrations. Data redundancy was assessed based on the SMILES representation. Duplicated molecules were identified and removed, giving 375 unique compounds, and the mean value for each measurement was calculated for each compound. To facilitate further analysis, the bioactivity data were converted to pChEMBL values using the following equation.

1 pChEMBL = −log_10_(Effective Value)

The next step is to generate the molecular representations. We opted for a Morgan fingerprint, also known as the extended-connectivity fingerprint (ECFP). Using a hashing function, ECFPs capture both local and global structural information, and encode these specifics and generalities into binary vectors.^25,26^ The Morgan fingerprint with radius 3 was calculated for each molecule using RDKit,^27^ generating 512 bits as the descriptors for each molecule.

### 2.2 Model construction and optimization

In order to construct ML models for regression, the Scikit package is utilized to develop ten regression models,^28^ including *K*-nearest-neighbour, Ridge, Lasso, elastic net, decision-tree, RF, extra-tree, adaboost, gradient boosting, and support vector regression (SVR). To ensure an unbiased evaluation of all models, a five-fold cross-validation approach was implemented to assess the performance and the generalization capability of the established models, where the dataset was segmented into five mutually exclusive subsets. In each fold, four subsets are used as the training set to develop the models while the remaining subset is used as the test set to evaluate the predictive performance of models. The use of predetermined random seeds guaranteed that each fold within the cross-validation procedure results in a controlled and reproducible data partition, enabling a rigorous assessment of model stability and consistency. Furthermore, adjusting the hyperparameters can improve the predictive power of a given model. Therefore, to systematically evaluate a range of potential parameter combinations, a grid search is applied on each model to identify the optimal model parameters by incorporating nested cross-validation.

### 2.3 Model evaluation and selection

Two metrics are used to evaluate the developed regressors, *i.e.*, the coefficient of determination *R*^2^ and the root mean squared error *e*_RMSE_.2
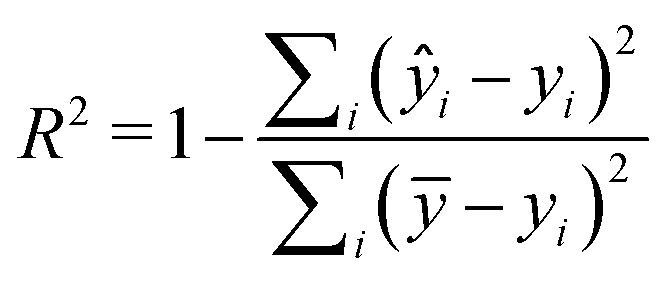
3
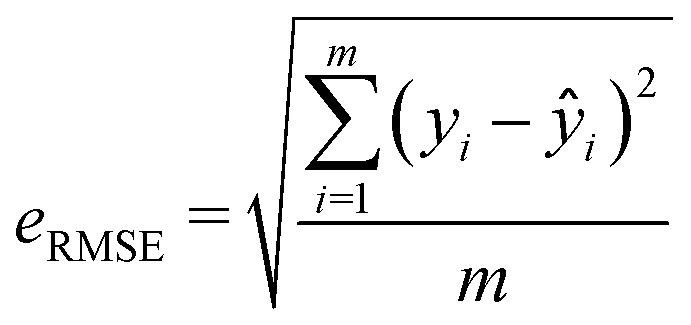
where *y*_*i*_, *ŷ*_*i*_ and *ȳ* represent the actual, expected, and mean pChEMBL values of compounds, and *m* is the number of samples.

The model validation process here was augmented by using two sets of labelled data, including external testing data comprising an additional 94 molecules derived from the latest literature, as well as a randomly selected 10% of the total dataset. To elaborate further, ten models were reconstructed using 90% of the reshuffled data from the entire dataset, resulting in 337 molecules, while the remaining 10% of data (38 compounds with diverse pChEMBL values) were combined with the aforementioned external data sourced from the literature (94 labelled compounds). Since this external verification set contains molecules of known pChEMBL value and it is never used to construct the models, the above simulated VS process allows a detailed examination of the model's predictions on a specific labelled dataset, evaluates the models on novel unseen molecules and detects the extent of model overfitting by quantifying the extent of the difference between the actual and the predicted activity value for this particular set. Complementing the five-fold cross validation mentioned above, this strategy, resembling a 10-fold cross-validation, providing an opportunity to scrutinize the model's predictions on a specific set of labelled data.

### 2.4 Virtual screening

After evaluating the models developed by the 10 methods, the re-trained regressor with 337 compounds that yielded the optimal performance is utilized to find prospective SOS1 inhibitors from various commercial chemical libraries. Specifically, we initiated the screening of the L-series commercial databases provided by the targetmol company (https://www.tsbiochem.com/), namely L1000, L4000, L6000, L5600, and L9200, which collectively encompass a total of 56 582 compounds. Additionally, we also screened the EGFR-related ChEMBL dataset due to the fact that SOS1 lead compounds originated from the EGFR project and the similarity of molecular structure between EGFR inhibitors and SOS1 inhibitors. During the VS, we employed the predicted pChEMBL value generated by our model to assess the potential inhibitory activity of each compound and molecules with a predicted pChEMBL value of 7 or higher were classified as ‘hits’. To broaden the exploration of chemical diversity, we further conducted screening within the expansive Chinese National Compound Library (Shanghai Institute of Materia Medica, Chinese Academy of Sciences, Shanghai, China), encompassing a substantial collection of 1 487 140 compounds. During this screening process, the top 200 compounds were selected for subsequent experimental validation without considering an activity threshold.

### 2.5 Protein–protein interaction assay

All the test compounds were provided by the CNCL (https://www.cncl.org.cn/). These compounds were supplied as stock solutions at a concentration of 1 or 5 mg mL^−1^ and dissolved in DMSO. The inhibitory activity was measured using the KRAS G12C/SOS1 PPI homogeneous time-resolved fluorescence (HTRF) assay at a final concentration of 10 μg mL^−1^, with **BI-3406** (MedChemExpress) as a positive control. For the IC_50_ measure of potentially active molecules, 1 or 5 mg mL^−1^ compound stock solution was selected to be gradient diluted at a final concentration of 0.5–50 μg mL^−1^. Briefly, the binding assay was performed in a white 384-shallow well microplate (PerkinElmer) with a final reaction volume of 20 μl in the binding domain detection buffer: 40 nM GST-tagged KRAS G12C protein (AntibodySystem), 20 nM His-tagged SOS1 protein (Cytoskeleton), 10 μM GTP, test compounds, MAb Anti GST-XL665 and MAb Anti-6HIS Tb cryptate Gold (PerkinElmer) were co-incubated at room temperature for 2 h. Subsequently, the HTRF signals were detected using a microplate reader (TECAN Spark®) with an excitation wavelength of 320 nm and emission wavelengths of 620 and 665 nm, respectively. The percentage inhibition was calculated by comparison with the buffer control group. The IC_50_ value was calculated by GraphPad Prism software in inhibitor *versus* normalized response (variable slope) mode.

### 2.6 Docking procedure

To identify the binding conformation of potential ligands in the active site, molecular docking was used to predict the interaction mode and binding energy between the selected ligands and SOS1 crystal structure (6SCM) from Protein Data Bank (PDB) using AutoDockVina4.^29^ Considering docking precision, semi-flexible docking was conducted, based on a stochastic docking procedure within the predefined docking box. The protein and ligand, formatted in PDBQT, were subjected to docking within a cubic box with specific coordinates: center_*x* = 7.540, center_*y* = −30.158, center_*z* = −43.243, and the dimension of 22.5 Å. AutoDockVina4 was used to reproduce nine distinct poses for each compound, but only the lowest energy constructs were considered as the best binding configuration. The analysis of protein-ligand interactions was conducted using protein–ligand interaction profiler (PLIP).^30^

### 2.7 *In silico* assessment of drug-like characteristics

Regarding the absorption, distribution, metabolism, excretion, toxicity (ADMET) properties, and pharmacochemical characteristics, the SMILES representation of the top molecule was submitted to an integrated online ADMET evaluation platform, ADMETlab2.0.^31^

## 3. Results and discussion

### 3.1 Dataset

Data collection from the ChEMBL database retrieved a total of 861 related molecules. Rigorous data curation protocols including data cleaning and duplicate removal resulted in 375 distinct molecules, which were used to generate molecular descriptors and construct the regression models. In order to model a QSAR well using regression models, it is desirable for the collection of molecules to encompass a broad range of inhibitory activities. Our data span a range of activities from 2.72 to 9.00. When the activity threshold is set to 7, a total of 214 compounds, representing 57% of the entire dataset, would be regarded as active, and this proportion is slightly higher than that of the inactive compounds.

### 3.2 t-SNE analysis

**Fig. 3:**
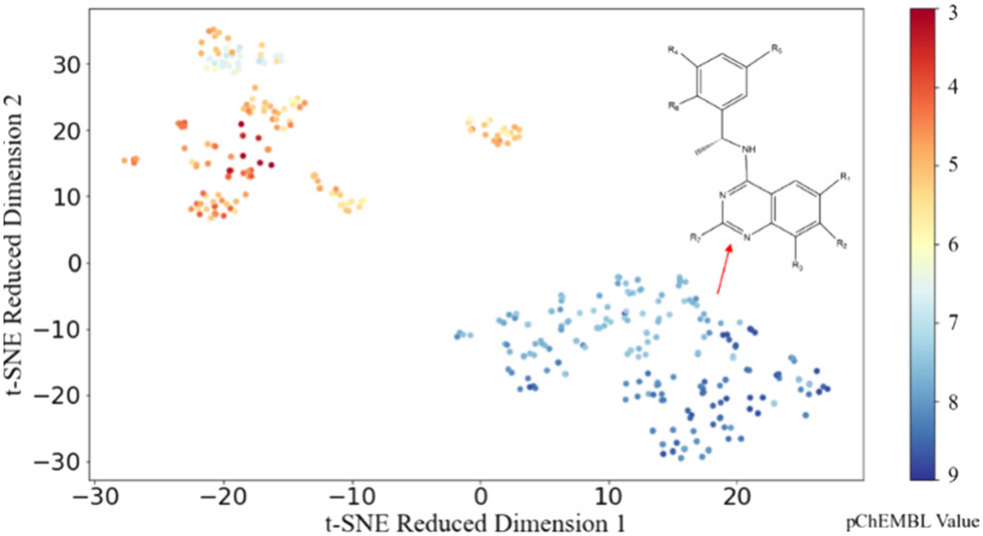
Visualization of the dataset using t-SNE. The colour bar represents pChEMBL values.

To explore the distribution of the dataset, t-distributed stochastic neighbour embedding (t-SNE), a data visualization method, is applied to the dataset with the ECFP molecular representation to visualize the spread and clustering of compounds. As shown in Fig. 3, two distinct clusters (orange and blue) are evident, and data points in the same cluster showed a small Euclidean distance between adjacent sets of data points. Such tight clusters with clear boundaries indicated the significant structural difference between active and inactive molecules. By examining the molecular structures in these two groups, we found that different from the orange cluster which contains different core structures, molecules in the blue cluster share similar core structures. As the molecules with similar outputs (pChEMBL values) cluster well in the 2D space obtained using t-SNE here, the analysis suggests that there are some potential patterns in the structures of active molecules that could be potentially recognized through ML models. Motivated by this t-SNE plot, we proceeded to construct regression models to discover the patterns embedded in the distribution of biologically active chemicals against SOS1.

### 3.3 Model validation and model selection

The statistical performance of the ten studied regression models is shown in Table 1. As indicated by their *R*^2^ and RMSE values, all these models showed relatively good prediction power (please refer to Table S1 in the ESI† for specific optimization parameters). Specifically, the RF-derived model outperformed others on the test set, achieving the lowest RMSE of 0.441 and the highest *R*^2^ of 0.918. Thus, the RF model was taken forward for the VS*.* Noticing that a relatively high standard deviation of 0.0596 for RF's test RMSE, which could indicate some potential variability in the prediction, to validate the model further, we carried out model reconstruction based on a subset of the whole data and conducted simulated VS using the residual dataset.

**Table 1:** The statistical performance of ten regression models is estimates using five-fold cross-validation. The standard deviation over the folds is shown in parentheses

Algorithm	Train *R*^2^	Test *R*^2^	Train RMSE	Test RMSE
Decision tree	0.985 (0.0023)	0.844 (0.0426)	0.191 (0.0154)	0.605 (0.0923)
Extra trees	0.994 (0.0012)	0.847 (0.0365)	0.119 (0.0134)	0.600 (0.0765)
AdaBoost	0.937 (0.0050)	0.899 (0.0176)	0.392 (0.0161)	0.489 (0.0548)
Ridge	0.989 (0.0014)	0.901 (0.0148)	0.164 (0.0103)	0.484 (0.0456)
SVR	0.989 (0.0017)	0.904 (0.0167)	0.165 (0.0127)	0.477 (0.0539)
*K*-neighbors	0.994 (0.0012)	0.905 (0.0169)	0.119 (0.0134)	0.475 (0.0498)
Gradient boosting	0.977 (0.0023)	0.910 (0.0201)	0.238 (0.0113)	0.463 (0.0596)
Lasso	0.943 (0.0033)	0.912 (0.0154)	0.373 (0.0109)	0.457 (0.0546)
Elastic net	0.952 (0.0029)	0.915 (0.0157)	0.343 (0.0102)	0.451 (0.0541)
Random Forest	0.983 (0.0014)	0.918 (0.0179)	0.205 (0.0084)	0.441 (0.0596)

The performance of ten reconstructed models using 90% of the reshuffled entire data may be found in the ESI† (Table S2), and the evaluation revealed that the predictive power of the RF model remained consistently superior. The simulated screening test on the labelled dataset showed that RF can successfully screen out 27 active molecules from 10% reserved dataset (38 molecules) and accurately identify the 55 out of 64 active molecules from the 94 SOS1-related compounds, utilizing a threshold based on the experimentally determined pChEMBL value of 7. As shown in the supporting information (Table S3†), the difference between predicted and actual activity values of compounds from the 10% reserved data is fairly small, with the maximum difference being 0.86. The reconstitution of models and their evaluation on a validation set provide insights into the model's uncertainty and an understanding of the model's overall performance, assessing the reliability, stability, and generalization capability of the selected model. In addition, when comparing the chemical structure of the external validation dataset molecules with that of the training set molecules, we found that the RF used for the simulated screening identified molecules with slightly different structure from the training set compounds and accurately predicted their activity. For instance, for the novel scaffold inhibitors reported by He *et al.*^15^ (as shown in **37** in Fig. 2.), our model could successfully pick out such tetracyclic quinazoline SOS1 inhibitors with a high predicted pChEMBL value of 8.29 against the actual value of 8.33, even though they were not included in the model development process. Therefore, we conclude that the RF regressor has a reliable predictive ability for SOS1 inhibitory activity, and it can also predict the activity of novel molecules with molecular backbones different from those in the training dataset.

### 3.4 Virtual screening of chemical libraries

The RF model was used as a filtering tool to predict promising candidates as potential SOS1 inhibitors from commercially available molecule databases. Employing a pChEMBL value 7 as a cut-off, a total of four hits from the L4000 small database and nine hits from EGFR ChEMBL were predicted to be active. A summary, including detailed information on these molecules resulting from the initial VS process, is presented in Tables 2 and 3.

**Table 2:** Known molecules screened from the L4000

Series	Compound ID	Structure	Predicted pChEMBL	Actual pChEMBL	Actual IC_50_ (nM)	Ref.
1	**BI-3406**	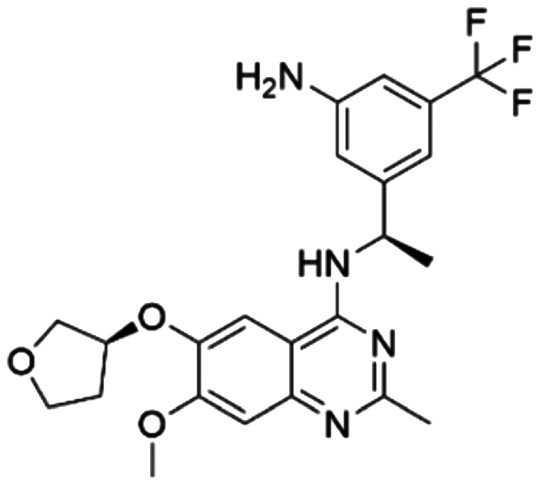	8.23	8.30	5	11
2	**I-37**	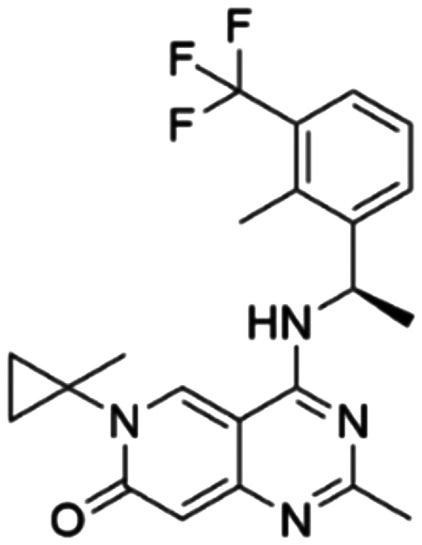	7.78	—	—	32
3	**I-49**	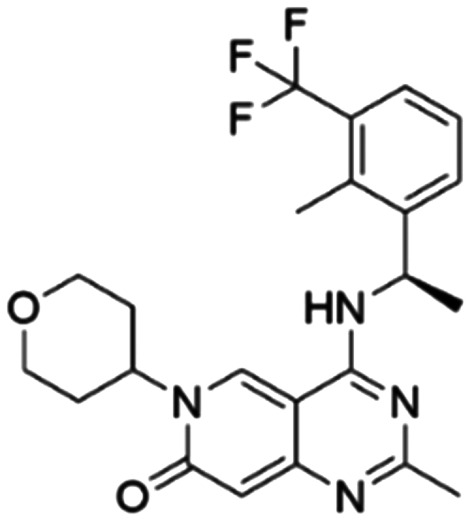	7.77	—	—	32
4	**BAY-293**	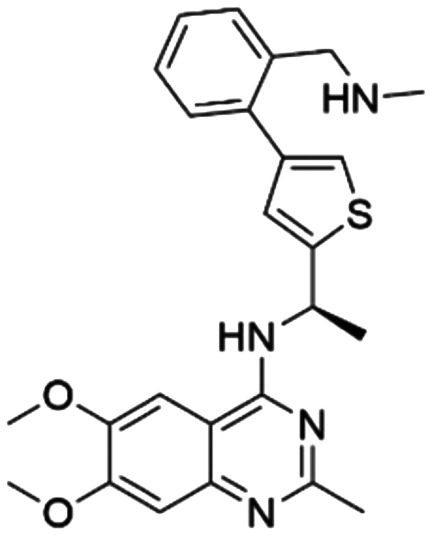	7.39	7.68	21	10

**Table 3:** Known molecules screened from the EGFR-related ChEMBL

Series	Compound ID	Structure	Predicted pChEMBL	Actual pChEMBL	Actual IC_50_ (nM)	Ref.
1	**CHEMBL5084571**	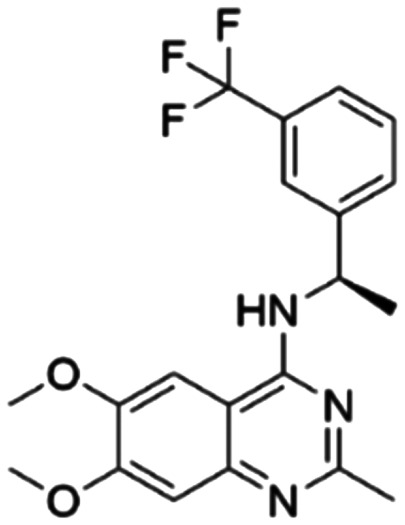	7.73	6.97	106	33
2	**CHEMBL4436731**	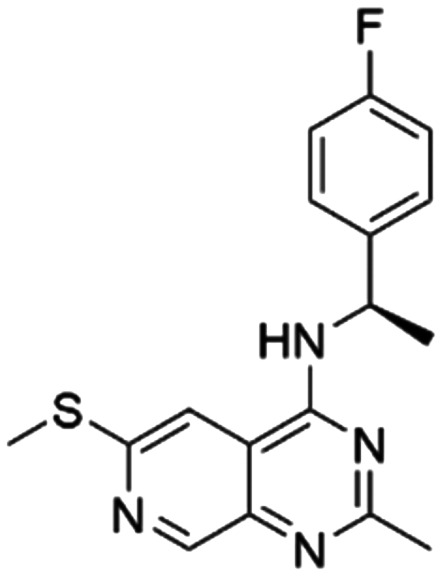	7.71	6.04	901	34
3	**CHEMBL5087955**	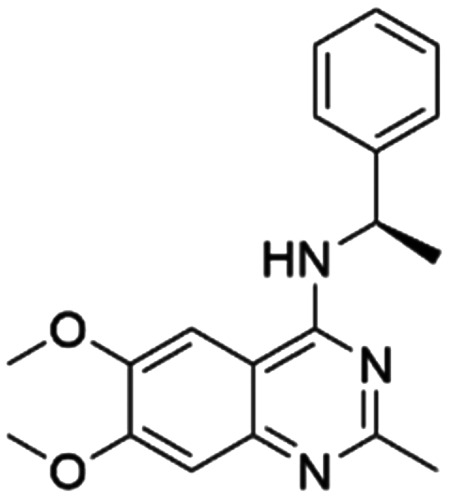	7.63	6.34	453	33
4	**CHEMBL4532624**	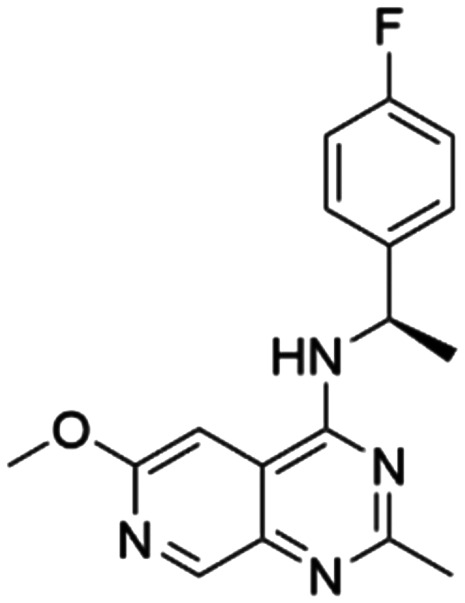	7.60	5.60	2510	34
5	**CHEMBL4445586**	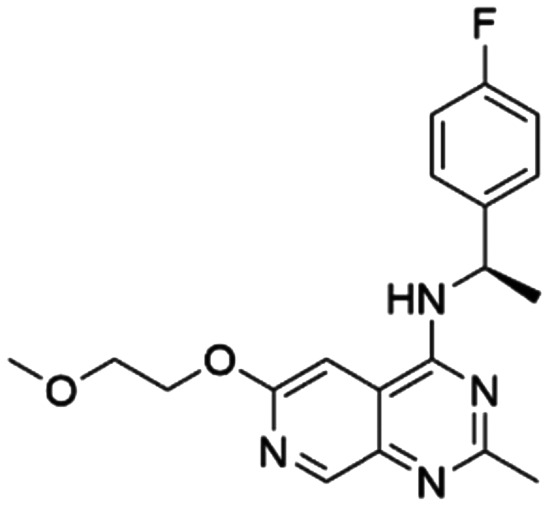	7.58	6.03	934	34
6	**CHEMBL4545109**	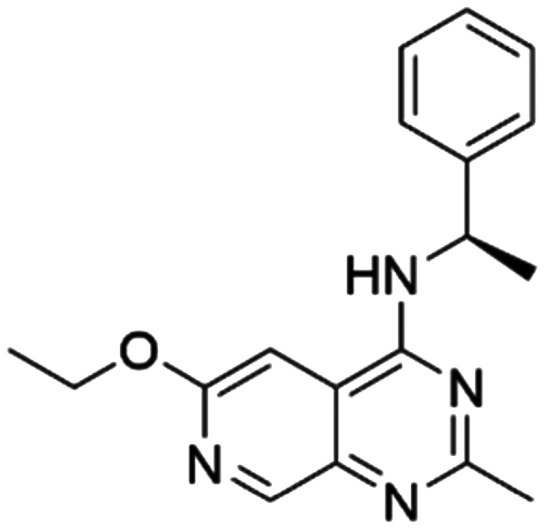	7.53	5.69	2060	34
7	**CHEMBL5085531**	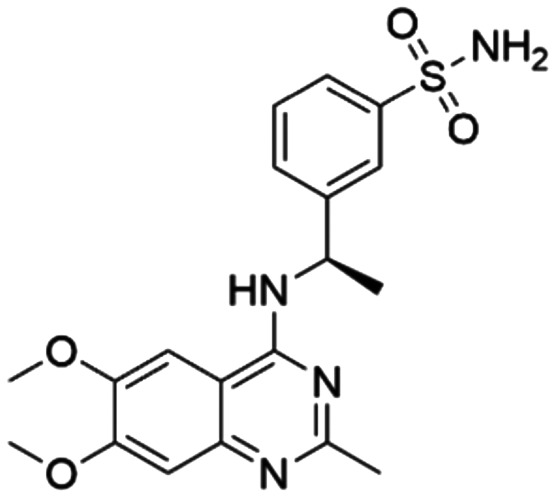	7.50	7.25	56	33
8	**CHEMBL4456281**	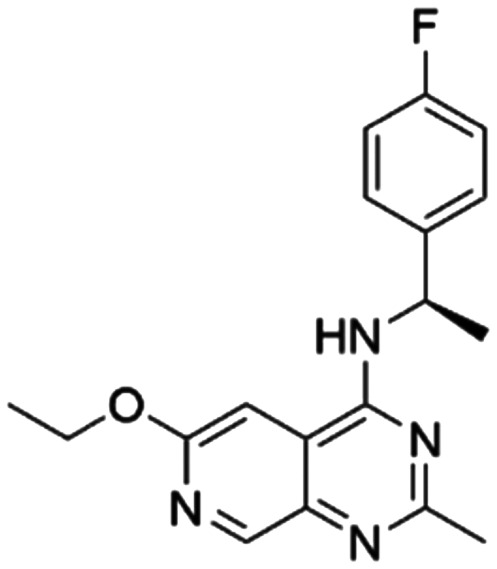	7.47	5.03	9300	34
9	**CHEMBL4463000**	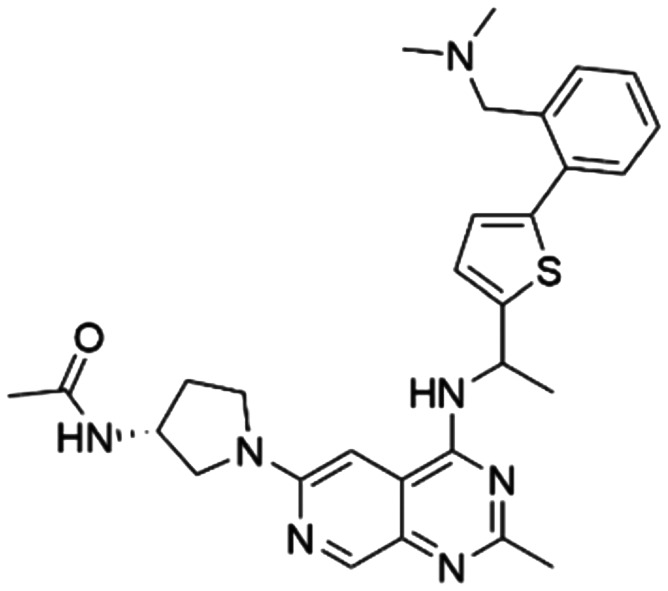	7.15	7.82	15	34

Among those screened molecules, **BI-3406** and **BAY-293** were reported to be SOS1 inhibitors.^10,11^ We found that four compounds' predicted pChEMBL values were very close to their actual ones, *i.e.*, 8.23 and 8.30 (5 nM) for **BI-3406**, 7.39 and 7.68 (21 nM) for **BAY-293**, 7.50 and 7.25 (56 nM) for **CHEMBL5085531**, and 7.15 and 7.82 (15 nM) for **CHEMBL4463000**, respectively. Noteworthily, we also identified eight compounds with novel inhibitor skeletons from those hits, with six of them sourced from the EGFR database (**CHEMBL4436731**, **CHEMBL4532624**, **CHEMBL4445586**, **CHEMBL4545109**, **CHEMBL4456281**, and **CHEMBL4463000**), exhibiting moderate activity as SOS1 inhibitors, and two of them, **I-37** and **I-49**, derived from an SOS1-related patent, although the patent does not provide quantitative activity information.^32^ Further structural analysis revealed that in contrast to known amino quinazoline inhibitors, those eight hits all have a pyrido pyrimidine scaffold, which did not appear in the training set. This structure has been designed and synthesized recently by Liu *et al.*^35^ as a novel potent SOS1 inhibitor pharmacophore. Furthermore, we compare the structural similarity of **I-37** and **I-49** with the training set based on the Tanimoto coefficient. The above two molecules did not share a high structural similarity, with Tanimoto coefficients of 0.439 for **I-37** and 0.433 for **I-49**. The limited similarity may be attributed to the absence of a quinazoline core, unlike the parent structure in the active compounds of the training set. In general, regression models are most likely to find highly analogous compounds or “me-too” hits, but using Tanimoto scores as a reference, the identified molecules were found to be structurally distinct from known SOS1 inhibitors, which revealed that the ML model in our study is capable of mining SOS1 inhibitors with novel chemical structures. Based on the insights from earlier investigations and exploring a broader chemical space, screening the large CNCL database helps us complement our VS efforts and identify novel SOS1 inhibitors. The top-ranked 200 promising compounds from the CNCL were subsequently submitted for further biological evaluation.

### 3.5 Biological activity test analysis

To validate our predictions, we measured experimentally the biochemical activity. During this screening process, we employed the HTRF PPI assay to evaluate the inhibitory effects of the compounds on the interaction between KRAS G12C and SOS1. We selected **BI-3406** as the positive control, and an IC_50_ of 31 nM was detected, which closely aligns with literature reports. It is worth noting that the *Z*′-score for this assay method is 0.95, exceeding the threshold of 0.5, indicating the reliability and accuracy of this assay. Out of the ranked top 200 compounds, we found nine of them exhibited modest inhibition at the final concentration of 10 μg mL^−1^ primary screening. Subsequently, we conducted activity confirmation assays at 25 μg mL^−1^ for these nine candidates (Table 4). The resulting four compounds with carboxylic acid backbone showed slightly stronger inhibitory activity against SOS1-KRAS PPI. Dose–response testing was performed on such potential hits, among which **CL01545365** was the most potent with an IC_50_ value close to 20 μg mL^−1^. Collectively, we identified a novel structural series capable of disrupting the SOS1-KRAS interaction.

**Table 4:** Information on the screened molecules: structural details, percentage inhibition (KRAS::SOS1), model-predicted pChEMBL value, and predicted docked energy by the VINA scoring function

Series	Compound ID	Structure	Inhibition rate at 25 μg mL^−1^ (%)	IC_50_ (μg mL^−1^)	Predicted pChEMBL	Docking energy (kcal mol^−1^)
1	**CL01545444**	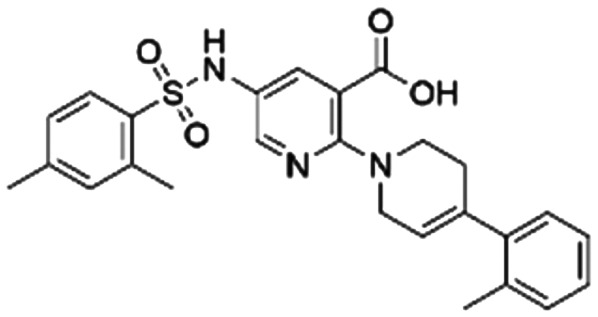	31.1 ± 1.4	49.2 (103 μM^a^)	5.83	−8.5
2	**CL01545464**	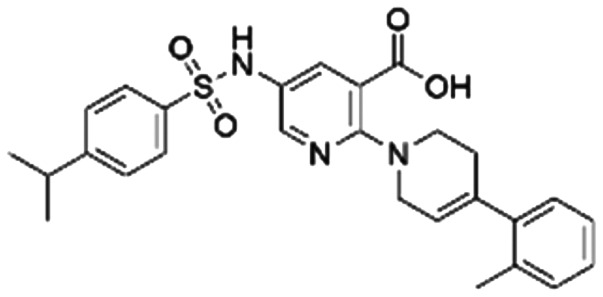	42.9 ± 3.4	32.6 (66.3 μM^a^)	5.96	−8.4
3	**CL01545365**	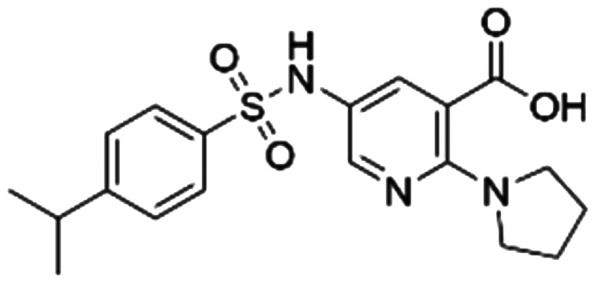	53.8 ± 1.6	20.9 (53.7 μM^a^)	5.81	−7.1
4	**CL01545355**	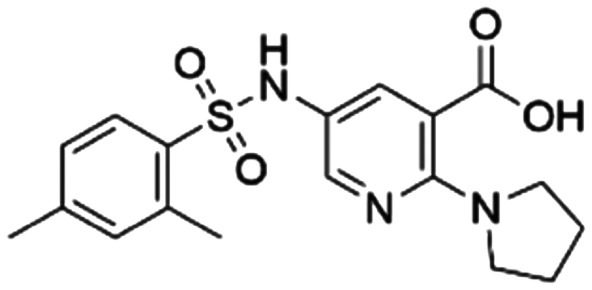	38.0 ± 3.3	35.5 (94.6 μM^a^)	5.80	−8.3
5	**CL00838284**	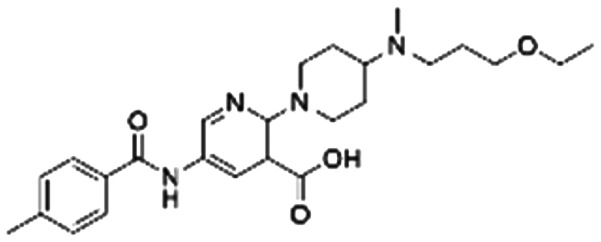	25.3 ± 0.3	ND^b^	5.83	−7.7
6	**CL01132463**	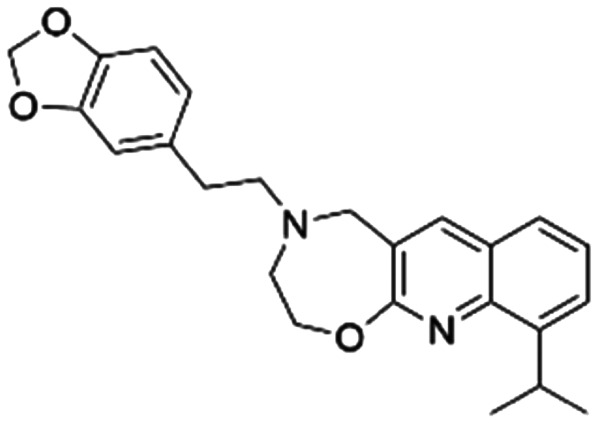	−6.5 ± 2.5	ND^b^	6.05	−8.8
7	**CL00838287**	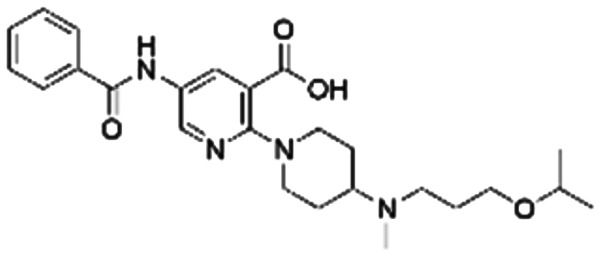	6.3 ± 6.6	ND^b^	5.85	−7.0
8	**CL00817024**	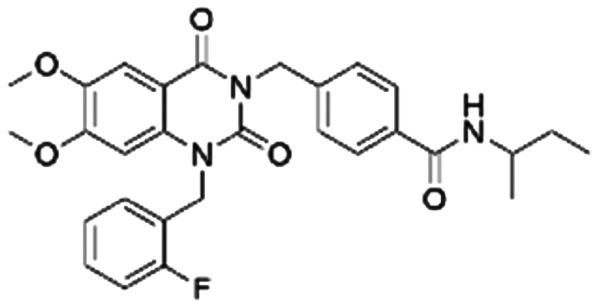	2.0 ± 0.8	ND^b^	5.82	−7.9
9	**CL01027021**	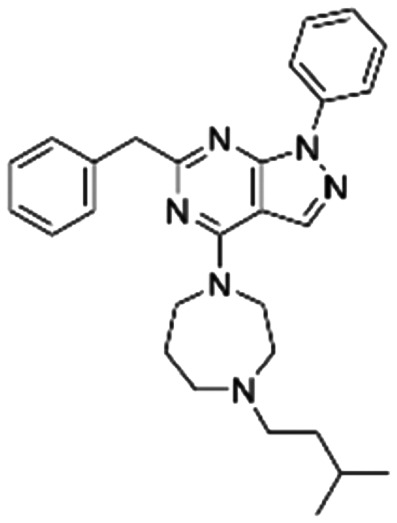	2.1 ± 1.4	ND^b^	6.07	−7.8

a The half-inhibitory concentration is calculated using the molecular weight of each compound.

b Not detected due to low activity.

### 3.6 Binding mode analysis of selected molecules

**Fig. 4:**
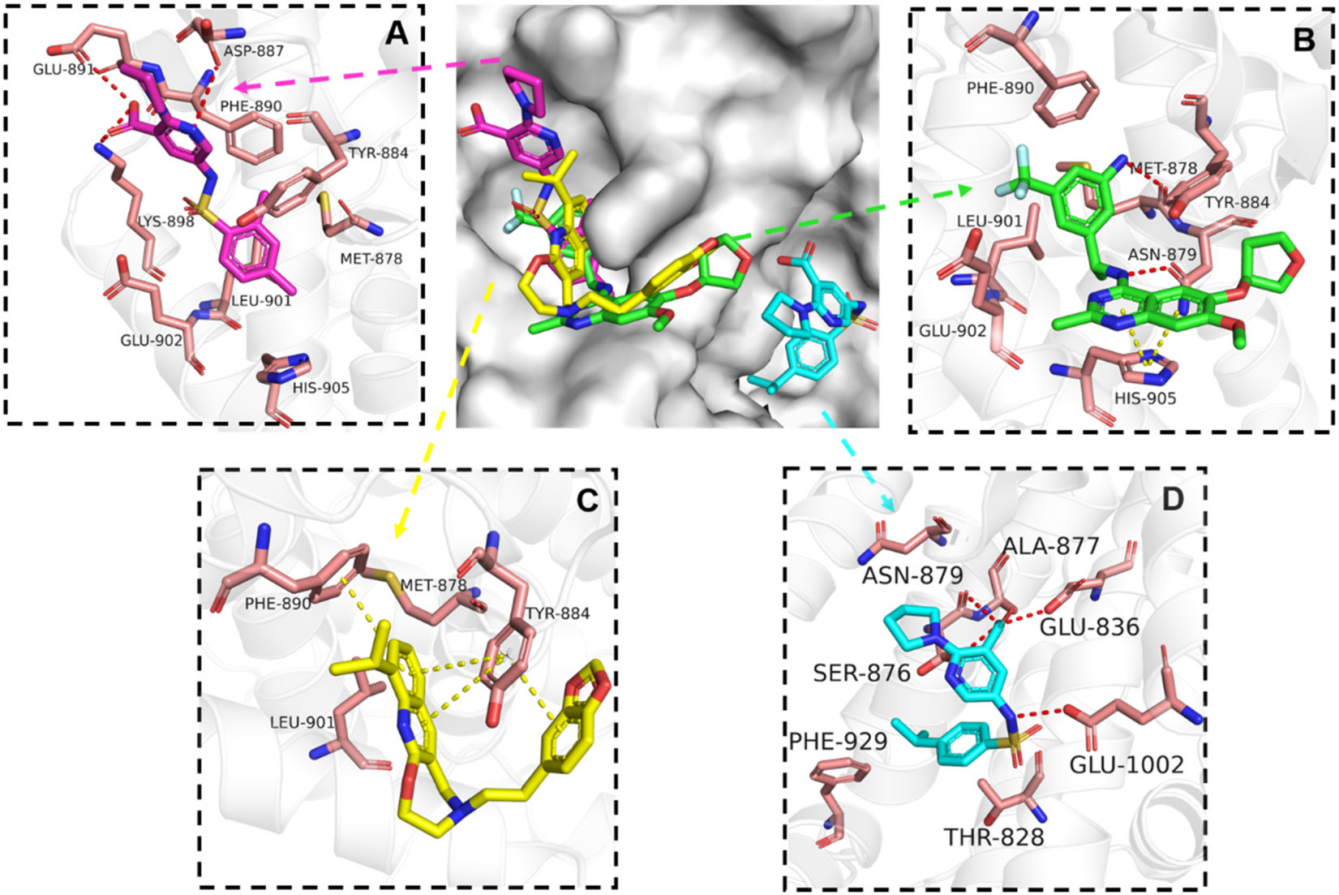
Interaction mode comparison of the hit compound and the known inhibitor against SOS1 protein (PDB:6SCM). (A) **CL01545355**; (B) **BI-3406**; (C) **CL01132463**; (D) **CL01545365**; the red dashed line represents the hydrogen bond interaction, and the yellow dashed line represents pi stacking; the protein-ligand interactions were analysed by PLIP (protein–ligand interaction profiler).^31^

The binding mode analysis of hit compounds and the target is carried out with respect to the known inhibitor, **BI-3406**. We observed that among the nine compounds selected from biological tests, two were confined within the same binding groove of the SOS1 crystal structure (**CL01132463** and **CL01545355**), while the remaining seven were found in binding pockets that are either identical to or in close proximity to the binding site of molecule **CL01545365** (See Table 2 for structural information, Fig. 4 for a comparison of docking modes, and Fig. S1† for the 2D receptor–ligand interaction).

The docking studies demonstrated that the predicted binding energy (−8.8 kcal mol^−1^) for compound **CL01132463** against 6SCM was higher than other hits. Such strong binding is mainly attributed to a series of pi stacking interactions between the ligand and residues Tyr884 and Phe890, which anchor it at the ligand-binding pocket of SOS1. Although those two residues form similar hydrophobic interactions, **BI-3406** employs a different anchoring method by establishing two pairs of key hydrogen bonds, including a key polar anchor between aniline N–H and Asn879, and interactions between amino substituent at the phenyl moiety and Met878. Moreover, the quinoline group of **CL01132463** protrudes into the hydrophobic pocket, forming another pi-stacking interaction with Phe890, while the oxazepino in the middle bends, allowing the ethyl group to interact well with Tyr884, and disrupt the R73 KRAS–Tyr884 binding like **BI-3406**, which was confirmed to be responsible for inhibitory function. Additionally, a series of hydrophobic interactions also contribute to the binding of **CL01132463**.

**CL01545355**, with the fourth-highest estimated binding affinity (−8.3 kcal mol^−1^), occupies the same binding site as **BI-3406** but exhibits a slightly different binding mode. Specifically, by engaging in hydrophobic interactions with Phe890, Tyr884, His905, and Leu901, the dimethylphenyl group is positioned in this hydrophobic area, and three hydrogen bonds are formed between the polar atoms on the niacin group and the surrounding polar protein residues, Asp887, Lys898, and Glu891. Notably, tetramine on pyrrolidine interacts with Asp887, and the carboxylate interacts with Lys898, forming two salt bridges. The hydrogen of the sulfonamide sulfur atom forms a pi-donor hydrogen bond with Phe890.

Interestingly, although **CL01545365** with the greatest potency has a similar scaffold structure to **CL01545355**, molecular docking suggested that it does not bind with the residues known to be involved in binding with known SOS1 inhibitors. Instead, by establishing three hydrogen bonds, docking predicts it to bind to the neighbouring pocket of **BI-3406** with a predicted binding energy of −7.7 kcal mol^−1^, as depicted in Fig. 4. The different binding energies (−7.7 kcal mol^−1^*versus* −8.3 kcal mol^−1^) observed between the two molecules may suggest distinct modes of interaction with the target, potentially indicating their accommodation within different pockets of 6SCM. The hydrogen atom on the carboxylic acid hydroxyl acts as a donor, forming hydrogen bond interactions with Glu836, Ser876 and Ala877, respectively, and a similar strong binding pattern can also be observed between the sulfonyl amide group and Glu1002 (1.79 Å). The dimethyl phenyl substituent forms two pi-alkyl interactions with Phe929, and other dominant nonpolar contributions including benzene ring binding to Thr828 and Ser876, were also identified as a factor for the anchoring of **CL01545365**. In comparison, the critical hydrogen bond formed by **BI-3406** with Asn879 was replaced by a hydrophobic interaction in **CL01545365**. Importantly, although compound **CL01545365** showed no interaction with Tyr884, which is believed to be crucial for the previous SOS1 inhibitory activity, biological activity testing revealed its ability to disrupt the interaction between SOS1 and KRAS at a micro-molar level. This implies that the binding site associated with this compound is likely to serve as a promising target for developing novel SOS1 inhibitors. Moreover, the docking suggested that **CL01545365** fills a shallow and wide pocket, which may reflect its relative low affinities in the predicted VINA scoring function. With the substantial structural difference from **BI-3406**, the **CL01545365** binding mode does not fully overlap with the **BI-3406** docked conformation. The comparable affinity reinforces the potential of such novel chemical structures for further development.

### 3.7 *In silico* evaluation of drug-like properties

The *in silico* ADMET of the top compound (**CL01545365**) was estimated to predict its pharmacochemical characteristics. As shown in Table S5,† all its properties fall within acceptable limits, except for the log *P* of 4.128, which is slightly larger than the maximum recommended value of 3. In addition, other drug-likeness parameters, including the MDCK permeability of 1.1 × 10^−5^ cm s^−1^ and volume of distribution of 0.245 L kg^−1^, are situated within the ideal range of 2 to 20 × 10^−6^ cm s^−1^ and 0.04 to 20 respectively. These predictions collectively suggest that molecule **CL01545365** possesses a relatively good pharmacokinetic profile and basically satisfies Lipinski's rule of five with only one violation, demonstrating its potential as a foundational candidate for the development of an SOS1 inhibitors. However, it is worth noting that while several ADMET properties of this molecule may not currently align with established drug-likeness criteria, further implementation of assays for physicochemical property evaluation (such as the Caco-2 cells assay for intrinsic permeability assessment, cardiotoxicity assay, *etc.*) should be undertaken to provide more accurate and pertinent information on this compound.

SOS1, a crucial protein within the RAS pathway, has recently garnered increasing attention as a promising strategy for treating RAS-driven cancers. However, the development of effective and selective SOS1 inhibitors remains a challenge. In our study, we employed LBVS combined with ML models to identify novel SOS1 inhibitors. We utilized the ChEMBL database for SOS1 as the target, which encompassed a diverse set of molecules with broad-ranging activity. Through the application of ML algorithms, such as SVR, RF Regressor, and Ridge Regressor, we evaluated and compared their performance in predicting the activity of SOS1 inhibitors. Among these algorithms, the RF model with the highest *R*^2^ and lowest RMSE in the test set demonstrated robustness and accuracy, making it the optimal choice for VS*.* Using the RF model, we screened commercially available compound databases. From the L4000 database and EGFR relevant dataset, we identified several inhibitory compounds reported in the literature, such as documented inhibitors **BAY-293** and **BI-3406**, and molecules previously reported in a patent, including **I-37** and **I-49**. Our findings also revealed that a subset of the above-screened molecules exhibited similar pharmacophore structures to the training set and positive controls but with distinct scaffolds, which highlights the potential of RF in discovering SOS1 inhibitors with different core structures. Building on the insights gained from the aforementioned results, we extended our screening efforts to the broader chemical space CNCL, encompassing more than 1.4 million compounds. From the pool of the 200 top-ranked molecules, we successfully identified nine candidate compounds exhibiting entirely distinct scaffolds from acknowledged SOS1 inhibitors, illustrating the novelty of the discovered inhibitors in this work. Represented by **CL01545365**, which has carboxylic acid as the skeleton, this type of molecule displayed moderate potency in the subsequent biological inhibitory activity assay: SOS1-mediated protein–protein interaction. Molecular docking indicated the presence of advantageous binding configurations, relatively strong binding energies and an unprecedented docking conformation, while an assessment of their suitability as drug candidates indicated their comparatively good drug-like properties, further supporting its potential as a SOS1 inhibitor. These compounds represent a prospective reservoir of novel molecular frameworks demonstrating efficacy against SOS1, thereby contributing to the design and refinement of related SOS1 inhibitors.

Several ongoing clinical trials are assessing the effectiveness of SOS1 inhibitors in cancer treatment. Specifically, these trials involve investigating **BI 1701963** as monotherapy and in combination with adagrasib (NCT04975256), or **BI 1823911** (NCT04973163), or MEK inhibitor trametinib (NCT04111458)^36^ or irinotecan (NCT0462742)^37^ for treating patients with KRAS mutated solid tumours. Thus, the utilization of **CL01545365** in conjunction with other chemotherapeutic drugs targeting the RAS oncogenic driver pathway aims to achieve enhanced therapeutic efficacy. Of course, there are some the limitations to our study. Data-driven AI models rely on a substantial volume of high-quality data as input for effective model training. Thus, a more extensive and diverse dataset of SOS1-related compounds would likely improve the predictive accuracy of the model. To mitigate the issue of data scarcity, a pre-trained model or transfer learning paradigm could be explored, and might enhance the model performance and reducing training time. We considered a single type of molecular representation for model construction; future work may incorporate other representations and fusion of the resultant composite features into deep learning models. Other future computational investigation will employ atomistic molecular dynamics simulation, to consider the pertinent dynamic interactions and conformational changes and go beyond the docking insights based on the static binding. Finally, experimental verification for the predicted drug-likeness properties of the lead compound is needed.

## 4. Conclusions

Overall, our research has revealed the potential of AI-driven LBVS as a valuable tool in discovering effective SOS1 inhibitors. By leveraging computational approaches, we have successfully identified and characterized lead compounds with new chemotype from databases, making a positive contribution to expanding the SOS1 inhibitor repertoire. These findings offer valuable insights for subsequent investigations and underscore new opportunities for therapeutic intervention in RAS-driven cancers.

## Abbreviations

The following abbreviations are used in this manuscript:

ADMET: Absorption, distribution, metabolism, excretion, and toxicity
AI: Artificial intelligence
CNCL: Chinese National Compound Library
ECFP: Extended-connectivity fingerprints
GDP: Guanosine diphosphate
GTP: Guanosine triphosphate
HTRF: Homogeneous time-resolved fluorescence
LBVS: Ligand-based virtual screening
ML: Machine learning
PDB: Protein Data Bank
PLIP: Protein–ligand interaction profiler
QSAR: Quantitative structure–activity relationship
RAS: Rat sarcoma virus
RF: Random forest
SMILES: Simplified molecular input line entry system
SOS1: Son of sevenless 1
SVR: Support vector regressor
t-SNE: t-distributed stochastic neighbour embedding
VS: Virtual screening

## Software and data availability

The data and code used in this study are available online at: https://github.com/cristinaduo/ML-for-SOS1.

## Conflicts of interest

There are no conflicts to declare.

## Supplementary Material

MD-015-D4MD00063C-s001
