# Supplementary material for: Discovery of novel SOS1 inhibitors using machine learning
Source: RSC Med Chem. 2024 Mar 15;15(4):1392–403. doi: 10.1039/d4md00063c (PMC11042245; doi:10.1039/d4md00063c)
Supplement: MD-015-D4MD00063C-s001 [file MD-015-D4MD00063C-s001.pdf]

Electronic Supplementary Material (ESI) for RSC Medicinal Chemistry.
This journal is © The Royal Society of Chemistry 2024

Supplementary Information

Discovery of Novel SOS1 Inhibitors Using Machine Learning

Lihui Duo,[a] Yi Chen,[c,e] Qiupei Liu,[a,c] Zhangyi Ma,[a] Amin Farjudian,[g] Wan Yong Ho,[d] Sze Shin Low,[a] Jianfeng Ren,[a,*] Jonathan D. Hirst,[b,*] Hua Xie,[c,e,f*] Bencan Tang [a,*]

[a] L. Duo, Q. Liu, Dr. Sze Shin Low, Dr. Jianfeng Ren, Dr. Bencan Tang
Nottingham Ningbo China Beacons of Excellence Research and Innovation Institute
Key Laboratory for Carbonaceous Waste Processing and Process Intensification Research of Zhejiang Province
Department of Chemical and Environmental Engineering, The University of Nottingham Ningbo China
199 Taikang East Road, Ningbo 315100 (P. R. China)

[b] Prof. Jonathan D. Hirst
School of Chemistry, University of Nottingham
University Park, Nottingham NG7 2RD (UK)

[c] Q. Liu, Yi Chen, Prof. Hua Xie
Division of Antitumor Pharmacology, State Key Laboratory of Drug Research, Shanghai Institute of Materia Medica, Chinese Academy of Sciences, 555 Zuchongzhi Road, 201203 Shanghai, China

[d] Dr. Wan Yong Ho
Faculty of Medicine and Health Sciences, University of Nottingham (Malaysia Campus),
Semenyih 43500, Malaysia

[e] Yi Chen, Prof. Hua Xie
University of Chinese Academy of Sciences, No.19A Yuquan Road, Beijing 100049, China

[f] Prof. Hua Xie
Zhongshan Institute for Drug Discovery, Shanghai Institute of Materia Medica, Chinese Academy of Sciences, Zhongshan Tsuihang New District, Zhongshan 528400, China

[g] Dr. Amin Farjudian
School of Mathematics, Watson Building, University of Birmingham, Edgbaston, Birmingham, B15 2TT UK

# Lihui Duo, Yi Chen, and Qiupei Liu contributed equally.

* E-mail addresses: Jianfeng.Ren@nottingham.edu.cn (Dr. Jianfeng Ren), jonathan.hirst@nottingham.ac.uk (Jonathan D. Hirst), hxie@simm.ac.cn (Prof. Hua Xie), Bencan.Tang@nottingham.edu.cn (Dr. Bencan Tang).

Supplementary Methods

1 Molecular dataset

The SMILES representations of all SOS1-related molecules from ChEMBL have been documented in distinct Excel files. Active and inactive molecules were distinguished based on a pChEMBL value of 7 as the threshold; visualizations of their molecular structures can be found in the folder named image within the GitHub repository: https://github.com/cristinaduo/ML-for-SOS1.

2 Background information on the machine learning algorithms

K-nearest-neighbor Regressor is a versatile non-parametric algorithm extensively employed in data analysis and prediction tasks, and it relies on the concept of feature similarity, operating under the assumption that data points with similar features tend to exhibit similar outcomes.[1] In practice, this algorithm identifies the nearest neighbors of a given data point within the training dataset based on their feature resemblance and employs a local interpolation technique to predict results.[1]

Ridge Regressor is a commonly used technique when dealing with multicollinearity, where independent variables exhibit high correlation.[2] By building upon linear regression, this algorithm introduces L2 regularization to add a penalty term to the regression coefficients, thus mitigating multicollinearity, and enhancing model stability.[2]

Lasso Regressor, akin to ridge regression, combats multicollinearity by incorporating

the L1 regularization to penalize the absolute value of regression coefficients, reduce variability and improve the accuracy of linear regression models.[3]

Elastic Net Regressor is a flexible algorithm that blends the best of Ridge and Lasso Regression methods, and it is well suited for handling the challenges of high dimensionality, primarily focusing on feature selection and regression.[4] Elastic Net combines the L1 (Lasso) and L2 (Ridge) regularization, which controls feature selection, prevents overfitting, and enhances the model robustness in the presence of multicollinearity. By fine-tuning the mixing ratio between these two regularization terms, Elastic Net adapts to various modeling requirements, making it a potent tool for regression tasks in diverse scenarios.[4]

Decision-tree Regressor is a tree-structured representation of decision-making processes that may categorize or predict continuous data, which splits the training data from the root node to the decision nodes.[5] Providing data type flexibility and legibility of resulting models, the Decision-tree Regressor could tackle the multi-class classification problems, but it suffers from potential noise and overfitting.

Random Forest Regressor is an ensemble approach to integrating multiple decision trees to tackle the problems of plagued bias and variance in decision trees, in order to improve the prediction performance and the model robustness.[6] By training each decision tree on a distinct data subset and introducing random feature selection for each split, Random Forest Regressor could effectively reduce correlations among individual trees, and leverage the predictions from multiple decision trees to enhance the ensemble's stability and accuracy.[6] Furthermore, its insight into feature importance aids

feature selection and model interpretation, making Random Forest Regressor a widely adopted and potent tool, especially valuable for tackling high-dimensional complex data across various applications.[6]

Extra-Tree Regressor is a powerful ensemble learning technique that builds a multitude of randomized decision trees through a meta estimator.[7] This approach introduces randomness by selecting random subsets of data and features during the tree-building process, which results in a more robust model with reduced overfitting and a higher prediction accuracy through ensemble-based averaging.[7]

Adaboost Regressor first fits a regressor on the original dataset and then fits subsequent copies of the regressor on the same dataset with the instance weights being changed in accordance with the error of the most recent prediction, to concentrate on challenging instances.[8]

Gradient Boosting Regressor is a powerful ensemble learning algorithm for regression that combines a collection of weak regression models such as decision trees in a sequential manner to progressively enhance the predictive performance by minimizing the loss function. This approach is particularly effective at capturing intricate nonlinear relationships while maintaining robustness, although it may require additional tuning and training time compared to alternative algorithms.[9]

Support Vector Regressor (SVR) finds a regression plane with the closest possible proximity to a subset of data points called support vectors, while allowing a controlled degree of deviation from these points.[10] SVR is able to capture the underlying patterns and relationships within the data while maintaining a balance between the model

complexity and the predictive accuracy.[10]

3 External validation dataset

The dataset has been documented in distinct Excel files on GitHub:

https://github.com/cristinaduo/ML-for-SOS1.

4 Similarity calculation

Similarities between molecules have been calculated from the Morgan fingerprints.

Supplementary Tables

Table S1. The optimal hyperparameters of 10 constructed models.

ML models	The optimal hyperparameters	Search space
Decision Tree	‘criterion’: friedman_mse, ‘min_samples_split’: 7	‘criterion’ in [squared_error, friedman_mse]; ‘min_samples_split’ ranges from 2 to 9.
Extra Tree	-	‘bootstrap’ in [True, False]; ‘min_samples_split’ ranges from 2 to 9.
Ridge	-	‘alpha’ in [0.001, 0.01, 0.1, 1, 10];
AdaBoost	‘learning_rate’: 1, ‘loss’: expomential	‘learning_rate’ in [0.001, 0.01, 0.1, 1]; ‘loss’ in [linear, square, expomential].
Gradient Boosting	‘min_samples_split’: 6	‘min_samples_split’ ranges from 2 to 9.
SVR	‘C’: 10, ‘gamma’: auto	‘C’ in [0.001, 0.01, 0.1, 1, 10, 20, 50, 100];

		‘gamma’ in [scale, auto].
K-Neighbors	‘algorithm’: ball_tree, ‘n_neighbors’: 9, ‘p’: 1	‘n_neighbors’ ranges from 2 to 10; ‘algorithm’ in [auto, ball_tree, kd_tree, brute]; ‘p’ in [1, 2].
Lasso	‘alpha’: 0.01, ‘selection’: random	‘alpha’ in [0.001, 0.01, 0.1, 1, 10]; ‘selection’ in [cyclic, random].
Elastic Net	alpha’: 0.01, ‘l1_ratio’: 0.7.	‘alpha’ in [0.001, 0.01, 0.1, 1, 10]; ‘l1_ratio’ in [0.2, 0.3, 0.4, 0.5, 0.6, 0.7, 0.8].
Random Forest	‘bootstrap’: False, ‘max_features’: sqrt, ‘min_samples_split’: 9	‘bootstrap’ in [True, False]; ‘max_features’ in [auto, log2, sqrt]; ‘min_samples_split’ ranges from 2 to 9.

Table S2. Algorithms mean performance for model validation using 90% data for

model refitting, and 10% reserved data for model verification: a measure of overfitting and external data validation.

**Algorithm**	**Train R^2**	**Test R^2**	**Train RMSE**	**Test RMSE**
Decision Tree	0.986(0.0019)	0.833(0.0549)	0.185(0.0133)	0.623(0.0998)
Extra Tree	0.996(0.0011)	0.833(0.0566)	0.099(0.0147)	0.622(0.0942)
Ridge	0.992(0.0014)	0.894(0.0187)	0.142(0.0118)	0.500(0.0462)
AdaBoost	0.940(0.0041)	0.898(0.0166)	0.389(0.0132)	0.491(0.0459)
Gradient Boosting	0.980(0.0019)	0.900(0.0242)	0.222(0.0103)	0.484(0.0687)
SVR	0.992(0.0015)	0.902(0.0182)	0.144(0.0131)	0.482(0.0495)
K-Neighbors	0.996(0.0011)	0.906(0.0184)	0.099(0.0147)	0.470(0.0461)
Lasso	0.946(0.0032)	0.910(0.0200)	0.367(0.0102)	0.462(0.0571)
Elastic Net	0.955(0.0024)	0.912(0.0189)	0.335(0.0085)	0.456(0.0535)
Random Forest	0.984(0.0015)	0.916(0.0145)	0.203(0.0088)	0.445(0.0437)

Table S3. The information of 10% data for model validation.

**Series**	**Compound ID**	**Structure**	**Predicted pChEMBL**	**Actual pChEMBL**
1	**CHEMBL4529467**	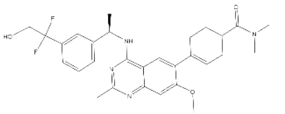	8.40	8.70

2	**CHEMBL4469357**	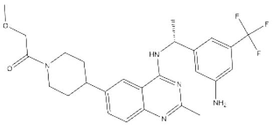	8.38	8.70
3	**CHEMBL4451252**	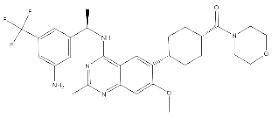	8.38	7.60
4	**CHEMBL4435672**	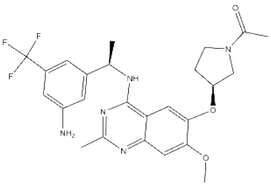	8.37	8.52
5	**CHEMBL4572922**	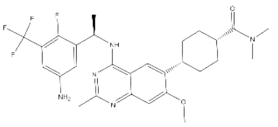	8.32	7.46
6	**CHEMBL4572076**	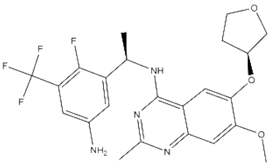	8.27	8.30
7	**CHEMBL4539190**	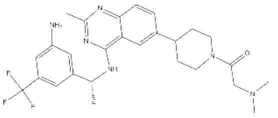	8.26	8.52
8	**CHEMBL4554249**	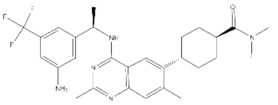	8.25	8.70
9	**CHEMBL4540213**	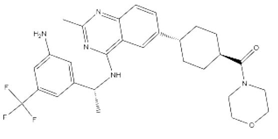	8.24	8.70
10	**CHEMBL4441820**	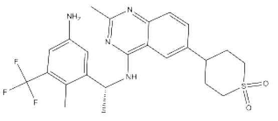	8.20	8.40
11	**CHEMBL4546387**	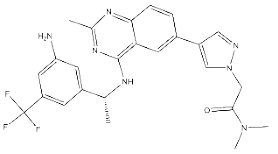	8.17	8.10
12	**CHEMBL4551748**	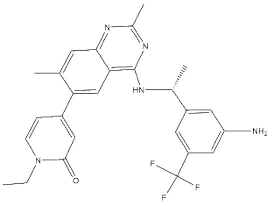	8.14	8.30
13	**CHEMBL4459389**	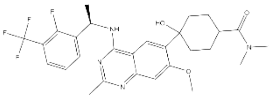	8.14	8.30

14	**CHEMBL4513254**	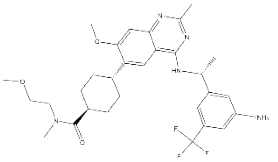	7.93	8.52
15	**CHEMBL4533487**	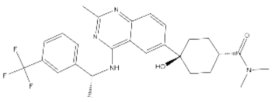	7.81	7.57
16	**CHEMBL4464090**	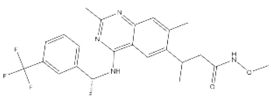	7.77	8.10
17	**CHEMBL4448274**	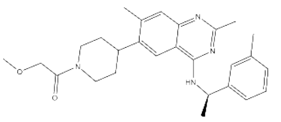	7.70	7.47
18	**CHEMBL4524954**	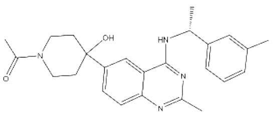	7.68	7.85
19	**CHEMBL4463184**	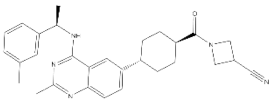	7.64	7.44
20	**CHEMBL4570224**	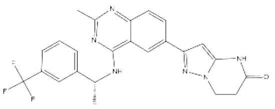	7.63	7.52
21	**CHEMBL4443395**	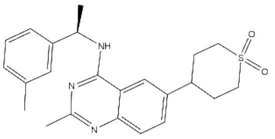	7.63	7.54
22	**CHEMBL4515122**	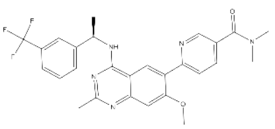	7.58	7.46
23	**CHEMBL4453639**	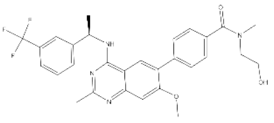	7.55	7.40
24	**CHEMBL4583745**	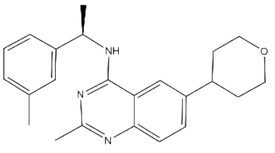	7.52	7.37
25	**CHEMBL4473214**	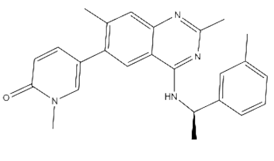	7.52	7.44
26	**CHEMBL4561965**	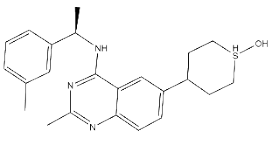	7.51	7.66

27	**CHEMBL4455684**	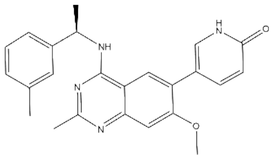	7.46	7.55

Table S4. Inhibition rate for selected carboxylic acid compounds at various concentrations in activity confirmation assays. Quantified data represents the mean ± SD from two independent biological replicates.

	**Inhibition rate (%)**				
**Concentration (μg/mL)**	**50**	**40**	**25**	**10**	**0.5**
**CL01545355**	61.6 ± 4.0	53.3 ± 0.5	38.0 ± 3.3	17.9 ± 3.3	-1.6 ± 0.2
**CL01545365**	72.3 ± 2.7	66.6 ± 0.1	53.8 ± 1.6	31.9 ± 2.8	0.8 ± 1.1
**CL01545444**	49.9 ± 0.5	45.5 ± 0.0	31.1 ± 1.4	14.5 ± 3.3	1.5 ± 1.1
**CL01545464**	60.5 ± 4.0	55.3 ± 3.2	42.9 ± 3.4	24.2 ± 1.4	3.4 ± 1.8

Table S5. Drug-likeness prediction of the molecule (**CL01545365**).

**Properties**		**Value**	**Optimal Range**		**Properties**	**Value**	**Optimal Range**
Physicochemical	Molecular Weight	389.5	100~600	Metabolism	CYP1A2 inhibitor	--	
Property	nHA	7	0~12		CYP1A2 substrate	+	

	nHD	2	0~7		CYP2C19 inhibitor	--	
	TPSA	99.600	0~140		CYP2C19 substrate	--	
	logS	-3.675	-4~0.5		CYP2C9 inhibitor	++	
	logP	4.128	0~3		CYP2C9 substrate	--	
	logD	1.210	1~3		CYP2D6 inhibitor	++	
Absorption	Caco-2 Permeability	-5.673	>-5.15		CYP2D6 substrate	--	
	MDCK Permeability	1.1 × 10−5	2−20 × 10−6		CYP3A4 inhibitor	-	
	Pgp-inhibitor	---			CYP3A4 substrate	++	
	Pgp-substrate	---		Excretion	CL	0.857	5~15
	HIA	---			T1/2	0.103	3
	F20%	---		Toxicity	LD50	1228.802	>500
	F30%	---			hERG Blockers	--	
Distribution	PPB	96.990%	<90%		H-HT	+	
	VD	0.245	0.04~20		DILI	+++	

	BBB Penetration	---			AMES	---	
	Fu	1.630%			SkinSen	---	

Molecular weight (MW) contains hydrogen atoms.

nHA: Number of hydrogen bonds acceptors.

nHD: Number of hydrogen bonds donors.

TPSA: Topological Polar Surface Area.

logS: log of the aqueous solubility.

logP: log of the octanol/water partition coefficient.

logD: logP at physiological PH 7.4.

Caco-2 Permeability: apparent Caco-2 cell permeability in log unit.

MDCK Permeability: apparent MDCK cell permeability in cm/s.

Pgp-inhibitor: possibility of being Pgp-inhibitor.

Pgp-substrate: possibility of being Pgp-substrate.

HIA: Human Intestinal Absorption.

F20%:20% bioavailability.

F30%:30% bioavailability.

PPB: Plasma Protein Binding.

VD: Volume Distribution.

BBB Penetration: Blood-Brain Barrier Penetration.

Fu: the fraction unbound in plasms.

CYP1A2 inhibitor: possibility of being inhibitor.

CYP1A2 substrate: possibility of being substrate.

CYP2C19 inhibitor: possibility of being inhibitor.

CYP2C19 substrate: possibility of being substrate.

CYP2C9 inhibitor: possibility of being inhibitor.

CYP2C9 substrate: possibility of being substrate.

CYP2D6 inhibitor: the possibility of being inhibitor.

CYP2D6 substrate: the possibility of being substrate.

CYP3A4 inhibitor: the possibility of being inhibitor.

CYP3A4 substrate: the possibility of being substrate.

CL: Clearance.

T1/2: half-life.

LD50: the dose amount of a tested molecule to kill 50% of the treated animals within a given period (mg/kg).

hERG Blockers: the probability of being active.

H-HT: Human Hepatotoxicity.

DILI: Drug-Induced Liver Injury.

AMES: Ames Mutagenicity; SkinSen: Skin sensitization.

Supplementary Figures

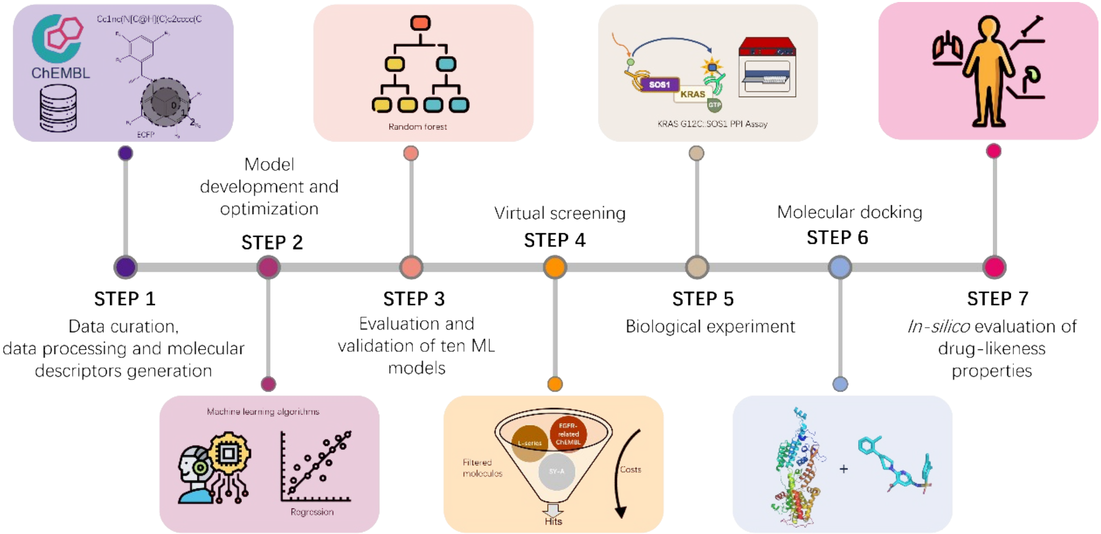

Figure. S1. Overview of the proposed pipeline for LBVS of SOS1. The framework consists of seven steps: 1) Raw data collection from the ChEMBL, data processing including curation, cleaning, and deduplicate, and molecular representation generation; 2) Model construction, and optimization; 3) Evaluating and adjusting the model parameters. 4) Virtual screening: molecules are searched from in-house libraries based on ML-based LBVS and hits are identified and ranked. 5) Biological experiment: KRAS G12C/SOS1 PPI Assay. 6) Molecular docking: hits and receptor interactions study; 7) *In-silico* evaluation of drug-likeness characteristics.

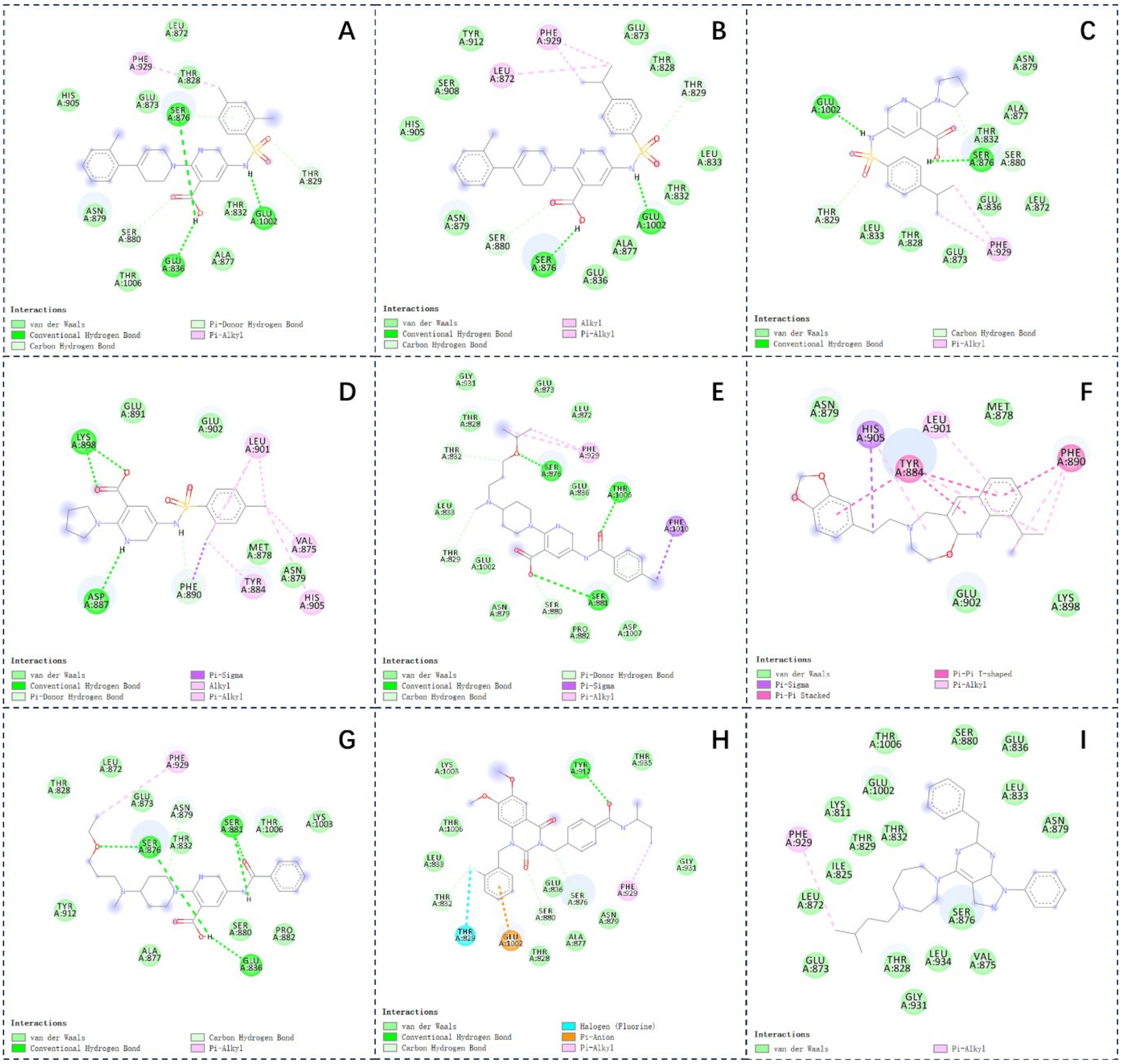

Figure. S2. 2D interaction mode of nine hit compounds with SOS1 protein (PDB:6CSM). (A) **CL01545444**; (B) **CL01545464**; (C) **CL01545365**; (D) **CL01545355**; (E) **CL00838284**; (F) **CL01132463**; (G) **CL00838287**; (H) **CL00817024**; (I) **CL01027021**. The receptor-ligand interaction was visualized using the BIOVIA Discovery Studio Visualizer (Version 2023, San Diego, Systèmes).

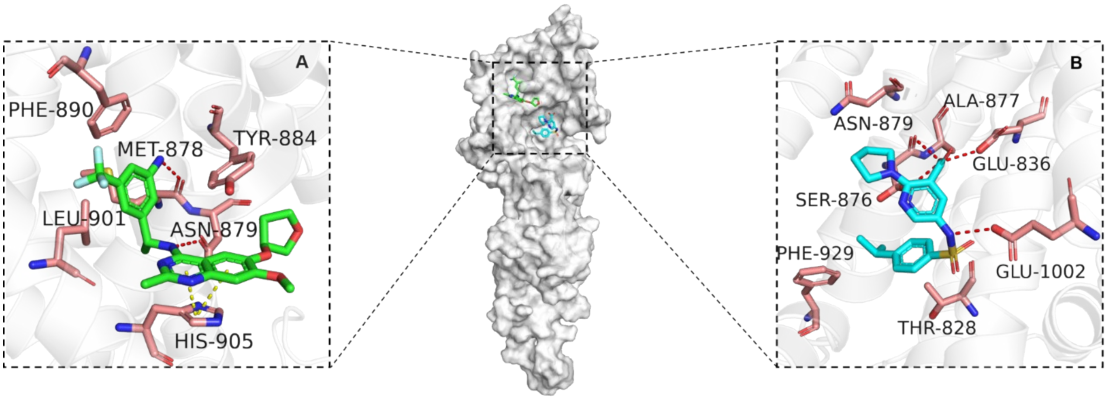

Figure. S3. Interaction mode comparison of the hit compound and the known inhibitor against SOS1 protein (PDB:6CSM). (A) **BI-3406**; (B) **CL01545365**; The red dashed line represents the hydrogen bond interaction; The protein-ligand interactions were analyzed by PLIP (Protein-Ligand Interaction Profiler).[11] .

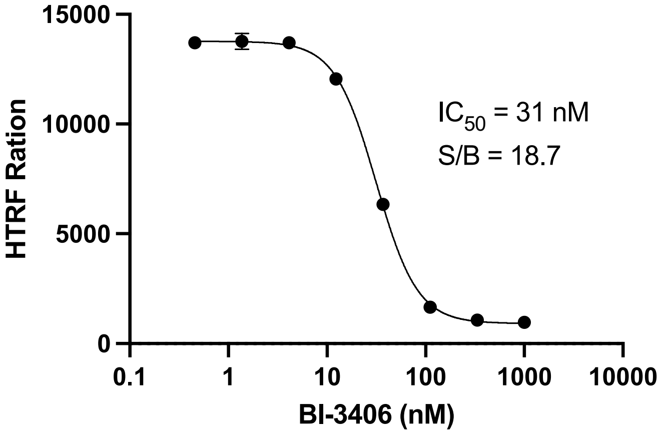

Figure. S4. The SOS1-KRAS PPI inhibitory activity of **BI-3406**.

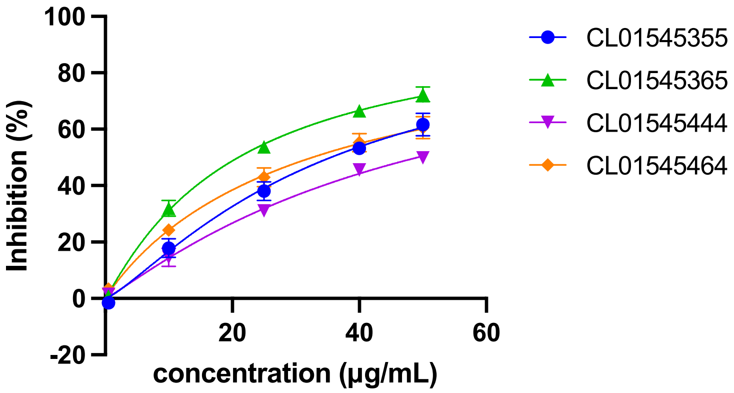

Figure. S5. IC_{50} curves of hits CL01545444, CL01545464, CL01545365, and CL01545355.

References

1 L. Devroye, L. Gyorfi, A. Krzyzak and G. Lugosi, *Ann. Statist.*, 1994, **22**, 1371–1385.

2 G. C. McDonald, *Wiley Interdisciplinary Reviews: Computational Statistics*, 2009, **1**, 93–100.

3 R. Tibshirani, *Journal of the Royal Statistical Society Series B: Statistical Methodology*, 1996, **58**, 267–288.

4 Z. Zhang, Z. Lai, Y. Xu, L. Shao, J. Wu and G.-S. Xie, *IEEE Transactions on Image Processing*, 2017, **26**, 1466–1481.

5 J. R. Quinlan, *Machine Learning*, 1986, **1**, 81–106.

6 L. Breiman, *Machine Learning*, 2001, **45**, 5–32.

7 P. Geurts, D. Ernst and L. Wehenkel, *Machine learning*, 2006, **63**, 3–42.

8 D. P. Solomatine and D. L. Shrestha, in *2004 IEEE International Joint Conference*

on Neural Networks, IEEE, 2004, **2**, 1163–1168.

9 J. H. Friedman, *Ann. Statist.*, 2001, **29**, 1189–1232.

10 A. Singh, N. Thakur and A. Sharma, in *2016 3rd International Conference on Computing for Sustainable Global Development (INDIACom)*, IEEE, 2016, pp. 1310–1315.

11 M. F. Adasme, K. L. Linnemann, S. N. Bolz, F. Kaiser, S. Salentin, V. J. Haupt and M. Schroeder, *Nucleic Acids Res.*, 2021, **49**, W530–W534.
